# The Common Gut Microbe *Eubacterium hallii* also Contributes to Intestinal Propionate Formation

**DOI:** 10.3389/fmicb.2016.00713

**Published:** 2016-05-19

**Authors:** Christina Engels, Hans-Joachim Ruscheweyh, Niko Beerenwinkel, Christophe Lacroix, Clarissa Schwab

**Affiliations:** ^1^Laboratory of Food Biotechnology, Institute of Food, Nutrition and Health, Department of Health Sciences and Technology, ETH ZurichZurich, Switzerland; ^2^Department of Biosystems Science and Engineering, ETH ZurichBasel, Switzerland; ^3^Research Informatics, Scientific IT Services, ETH ZurichBasel, Switzerland; ^4^SIB Swiss Institute of BioinformaticsBasel, Switzerland

**Keywords:** *Eubacterium hallii*, propionate, propanediol, reuterin, gut microbe

## Abstract

*Eubacterium hallii* is considered an important microbe in regard to intestinal metabolic balance due to its ability to utilize glucose and the fermentation intermediates acetate and lactate, to form butyrate and hydrogen. Recently, we observed that *E. hallii* is capable of metabolizing glycerol to 3-hydroxypropionaldehyde (3-HPA, reuterin) with reported antimicrobial properties. The key enzyme for glycerol to 3-HPA conversion is the cobalamin-dependent glycerol/diol dehydratase PduCDE which also utilizes 1,2-propanediol (1,2-PD) to form propionate. Therefore our primary goal was to investigate glycerol to 3-HPA metabolism and 1,2-PD utilization by *E. hallii* along with its ability to produce cobalamin. We also investigated the relative abundance of *E. hallii* in stool of adults using 16S rRNA and *pduCDE* based gene screening to determine the contribution of *E. hallii* to intestinal propionate formation. We found that *E. hallii* utilizes glycerol to produce up to 9 mM 3-HPA but did not further metabolize 3-HPA to 1,3-propanediol. Utilization of 1,2-PD in the presence and absence of glucose led to the formation of propanal, propanol and propionate. *E. hallii* formed cobalamin and was detected in stool of 74% of adults using 16S rRNA gene as marker gene (*n* = 325). Relative abundance of the *E. hallii* 16S rRNA gene ranged from 0 to 0.59% with a mean relative abundance of 0.044%. *E. hallii* PduCDE was detected in 63 to 81% of the metagenomes depending on which subunit was investigated beside other taxons such as *Ruminococcus obeum, R. gnavus, Flavonifractor plautii*, *Intestinimonas butyriciproducen*s, and *Veillonella* spp. In conclusion, we identified *E. hallii* as a common gut microbe with the ability to convert glycerol to 3-HPA, a step that requires the production of cobalamin, and to utilize 1,2-PD to form propionate. Our results along with its ability to use a broad range of substrates point at *E. hallii* as a key species within the intestinal trophic chain with the potential to highly impact the metabolic balance as well as the gut microbiota/host homeostasis by the formation of different short chain fatty acids.

## Introduction

The mammalian intestine is densely populated by trillions of bacteria belonging to an estimated 500–1,000 species that are predominantly classified as *Firmicutes* and *Bacteroidetes*. Under conditions of health, host and microbiota live in a mutualistic relationship. The host supplies energy sources through ingestion of food and secretion of metabolites from the epithelium, and the key roles of the microbiota are the breakdown of nutrients such as dietary and host derived glycans, the synthesis of hormones and vitamins, and colonization resistance to pathogens (Chassard and Lacroix, 2013; Lee and Hase, 2014). Short chain fatty acids (SCFA), such as butyrate, acetate and propionate, formed during microbial fermentation of dietary or host derived carbohydrates, supply the host with energy but also mediate interactions with the immune system (Louis et al., 2014).

Trophic interactions of microbes are required for glycan degradation and fermentation. Complex carbohydrates derived from diet or host (mucins) are degraded by fibrinolytic or mucinolytic bacteria, further metabolism of the hexoses and pentoses yields acetate, propionate, butyrate, formate, succinate, and lactate (Chassard and Lacroix, 2013; Louis et al., 2014). Propionate and butyrate are final products of bacterial metabolism that can be taken up by the host. Succinate can be further metabolized (for e.g., by *Phascolarctobacterium succinatutens*) to form propionate. Formate is a substrate for methanogenic archaea. Lactate can be used by many species. For example *Veillonella* forms mainly acetate and propionate from lactate. Members of the *Lachnospiraceae* such as *Eubacterium hallii* and *Anaerostipes* spp. utilize acetate and lactate to produce butyrate and also hydrogen (Duncan et al., 2004).

Due to its ability to utilize the intermediate lactate, *E. hallii* is considered an important microbe in regard to intestinal metabolic balance as lactate accumulation has been associated with several intestinal diseases and malabsorption (Hove et al., 1994; Duncan et al., 2004). However, the substrate utilization spectrum is not limited to lactate and acetate, as *E. hallii* also forms butyrate from glucose (Duncan et al., 2004), but does not grow on or utilize more complex oligo- and polysaccharides (Scott et al., 2014).

**FIGURE 1:**
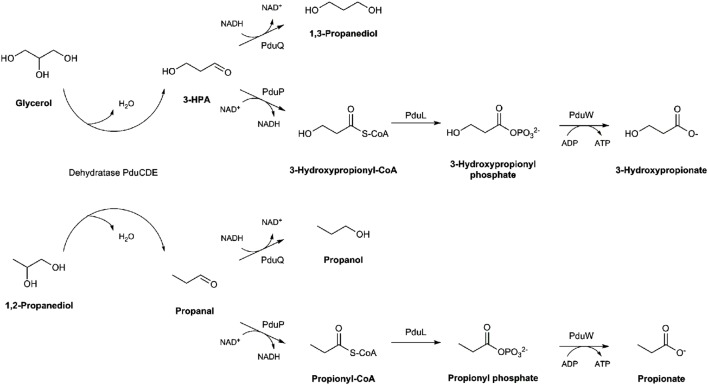
**Glycerol and 1,2-propanediol metabolic pathways**.

Recently, we observed that *E. hallii* is capable of metabolizing glycerol to 3-hydroxypropionaldehyde (3-HPA) which in aqueous solution exists in a multi-compound system called reuterin (Engels et al., 2016). Reuterin has antimicrobial activity against Gram-positive and Gram-negative bacteria, fungi and yeast, probably by causing oxidative stress through interaction with intracellular glutathione (Schaefer et al., 2010; Vollenweider et al., 2010). Reuterin formation has previously been reported for members of the genera *Klebsiella, Enterobacter, Citrobacter, Clostridium*, and *Lactobacillus* (Vollenweider and Lacroix, 2004). The key enzyme that catalyzes glycerol to 3-HPA conversion is the glycerol/diol dehydratase PduCDE (Morita et al., 2008). A second substrate of this enzyme is 1,2-propanediol (1,2-PD) which can be further metabolized to propanol or propionate thereby generating one ATP (Gänzle, 2015; **Figure 1**). Glycerol/diol dehydratases are cobalamin (vitamin B12)-dependent enzymes. In *Lactobacillus reuteri*, a cluster of 58 genes is involved in the biosynthesis of reuterin and cobalamin (Morita et al., 2008). The *pdu* operon harbors genes encoding for the glycerol/diol dehydratase as well as enzymes which enable the formation of propionate if 1,2-PD is present as substrate; genes required for cobalamin synthesis are located within the *cbi-cob-hem* operon (Santos et al., 2008). We observed that *E. hallii* harbored an almost complete *pdu* and *cob* loci, however, in comparison to *L. reuteri*, gene synteny of the *pdu-cbi-cob-hem* cluster was not preserved (Engels et al., 2016; **Supplementary Figure S1**). *E. hallii* thus possesses the genomic potential to metabolize glycerol and 1,2-PD, and to synthesize cobalamin, however, phenotypic confirmation is missing.

Glycerol and 1,2-PD are readily available in the gastrointestinal tract as they are derived from the metabolism of triglycerides, or fucose and rhamnose, respectively. Latter are building blocks of dietary (for e.g., human milk oligosaccharides) or host derived glycans (mucin; Podolsky, 1985; Casas and Dobrogosz, 2000). The ability of *E. hallii* to metabolize glycerol and 1,2-PD could result in a competitive advantage by the formation of the antimicrobial compound reuterin or the production of propionate, respectively. Therefore, our primary goal was to investigate the glycerol and 1,2-PD metabolism of *E. hallii.* We also investigated the relative abundance of *E. hallii* in adult stool samples derived from the human genome project (HMP, Human Microbiome Project Consortium, 2012) using 16S rRNA and *pduCDE* gene screening to determine the contribution of *E. hallii* to intestinal propionate formation.

## Material and Methods

### Bacterial Strains and Culture Conditions

Stab cultures of *E. hallii* DSM 3353 and DSM 17630 were frozen at -20°C in YCFA agar (1.5% (w/v) agar, **Supplementary Table S1**) and used as stock cultures. For each experiment, a fresh agar stock was thawed; 1 ml of liquid YCFA medium was added and thoroughly shaken before being transferred to 8 ml liquid YCFA medium. After incubation at 37°C for 72 h, the culture was transferred at least once to fresh YCFA broth before the experiment. The YCFA medium was prepared as described by Duncan et al. (2004) with slight modifications and contained approximately 33 mM acetate (mYCFA, **Supplementary Table S1**). All components except L-cysteine were solubilized in deionized water, pH was adjusted to pH 7.6 with NaOH. After boiling and a color change from blue to pink, L-cysteine (0.01%, w/v) was added and tubes were autoclaved. Unless otherwise stated, mYCFA containing 180 mM glucose was used to routinously cultivate *E. halli.* Stock cultures of *L. reuteri* DSM 20016, *L. rossiae* DSM 15814 and *L. delbrueckii* subsp. *lactis* DSM 20355 were maintained at -80°C in 30% glycerol. Lactobacilli were routinously grown in MRS at 37°C for 24 h.

### Growth in the Presence of Different Substrates

Growth kinetics were assessed in mYCFA medium supplied with glucose, glycerol, lactate or 1,2-PD as summarized in **Table 1.** Hungate tubes containing 9 ml mYCFA were inoculated with 0.5 ml of overnight cultures of *E. hallii* strains DSM 3353 or DSM 17630. Samples were taken after 0, 3, 6, 9, 12, and 24 h of incubation for substrate and metabolite analysis. Bacterial growth was evaluated by measuring the optical density at 600 nm (OD_600_). Growth in the presence of glucose was investigated in six independent replicates, all other substrates were tested in independent triplicates.

**Table 1:** Substrates (carbon sources) added to mYCFA medium.

Substrate	Abbreviation	Amount (mM)
Glucose	mYCFA_glc	70
Glycerol	mYCFA_gly	120
Glucose+glycerol	mYCFA_glc_gly	70 + 120
Lactate	mYCFA_lac	40
Lactate+glycerol	mYCFA_lac_gly	40 + 120
1,2-PD	mYCFA_pd	80
1,2-PD+glucose	mYCFA_pd_glc	70 + 80


*All media contained approximately 33 mM of acetate.*

### Analysis of Glycerol and Its Conversion Products, Glucose Consumption, and SCFA Formation

Glucose consumption was measured using high performance liquid chromatography (Merck-Hitachi, Darmstadt, Germany) equipped with an Aminex HPX-87H column (300 mm × 7.8 mm; BioRad) and a refractive index detector (HPLC-RI). Samples were centrifuged at 13 000 g for 5 min at 4°C. Supernatants (40 μL injection volume) were eluted with 10 mM H_2_SO_4_ at a flow rate of 0.6 ml min^-1^ at 40°C. Glucose was quantified using external standards.

Glycerol, 1,2-PD, 1,3-propanediol (1,3-PD), 3-HPA, 3-hydroxypropionate, propanal, and propanol were quantified with ion chromatography with pulsed amperometric detection (IC-PAD) on a Thermo Scientific ICS-5000^+^ system equipped with a quaternary gradient pump, a thermo autosampler, and an electrochemical detector with a cell containing an Ag/AgCl reference electrode and a disposable thin-film platinum working electrode tempered at 25°C. Analytes were separated on a Thermo Scientific IonPac ICE-AS1 4 × 250 mm ion-exclusion column with guard column operated at 30°C using isocratic conditions (0.1 M methanesulfonic acid; 0.2 mL min^-1^) for 36 min. The injection volume was 10 μL. Electrochemical data was obtained using a triple potential waveform consisting of regeneration/detection, oxidation and reduction potentials: *E*_1_ = 0.3 V (*t*_1_ = 0.31 s), *E*_2_ = 1.25 V (*t*_2_ = 0.34 s, *t*_int_ = 0.02 s), *E*_3_ = -0.4 V (*t*_3_ = 0.39 s). Currents were measured and integrated with respect to time (*t*_int_).

Short-chain fatty acids (SCFAs) acetate, propionate, butyrate, formate, and lactate were quantified with IC with suppressed conductivity detection on the previously described ICS-5000^+^ system using external standards. Analytes were separated on a IonPac AS11-HC 4 × 250 mm column with guard column operated at 30°C using the following gradient conditions at 1.5 ml min^-1^ using an eluent generator: 1.5 mM KOH, 0–6 min; 1.5–35 mM KOH, 6–21 min; 35–60 mM KOH, 21–26 min; 60 mM KOH, 26–27 min; 60–1.5 mM KOH, 27–28 min followed by appropriate re-equilibration. The injection volume was 10 μL.

### Cobalamin Production

Cobalamin formation was determined as described by Langa et al. (2013) using the cobalamin auxotrophic *L. delbrueckii* subsp. *lactis* DSM 20355 as reporter strain. Known cobalamin producers *L. reuteri* DSM 20016 and *L. rossiae* DSM 15814 (Sriramulu et al., 2008; De Angelis et al., 2014) as well as vitamin B12-deficient assay medium with added vitamin B12 served as positive controls. Vitamin B12-deficient assay medium without any cellular extract was used as negative control. Bacterial growth was determined by measuring the optical density at 600 nm after incubation at 37°C for 24 h. Biological duplicates of the experiment were performed.

### Minimum Inhibitory Concentrations of Reuterin toward the Two Strains of *E. hallii*

Minimum inhibitory concentrations (MICs) of reuterin toward *E. hallii* strains DSM 3353 and DSM 17630 were determined using serial dilutions of a reuterin solution containing 186 mM 3-HPA added to mYCFA. Hereby, *L. reuteri* DSM 20016 was cultured in MRS medium at 37°C before washed cells were suspended in glycerol solutions; reuterin was isolated from resulting solutions as described previously (Vollenweider et al., 2003). mYCFA containing reuterin was inoculated with overnight cultures of *E. hallii* strains at 5% (v/v). Bacterial growth was determined by measuring the OD_600_ after incubation at 37°C for 24 h. A sigmoidal equation was used to fit the data (Sigma Plot version 12); the inflection point of the resulting curves represented the MIC_50_ value quantified as 3-HPA which was defined as the lowest concentration that reduced final OD_600_ to 50% compared to controls without inhibition. The experiment was performed in four independent replicates for each strain.

### 16S rRNA Gene Library Screening

The 325 stool 16S rRNA gene sequences datasets from the HMP (Human Microbiome Project Consortium, 2012) were downloaded from ftp://public-ftp.hmpdacc.org/HM16STR (SRA: SRP002395). The datasets contain ∼6 million bacterial 16S rRNA sequences from the variable regions V1–3, V3–V5, and V6–9. Sequences are on average 406 bases long. The smallest dataset contained 250 sequences, the largest contained 140,179 sequences with an average number of 18,370 sequences per dataset.

Screening was performed using 16S rRNA gene sequences extracted from *E. hallii* DSM 17630 and DSM 3353. Version 123 of the Silva 16S rRNA database (Quast et al., 2013) served as additional reference.

*Eubacterium hallii* DSM 17630 and DSM 3353 16S rRNA gene reference sequences were aligned against the Silva database using MALT’s dynamic programming semi-global alignment algorithm (Huson, 2014) to generate a list of potential false positives. Sequences in Silva that aligned at an identity larger than 95% and did not originate from *E. hallii* were labeled false positives.

Data from the 325 samples were then aligned against the *E. hallii* reference database using the dynamic programming. Only alignments with a minimum identity of 97% were reported. Sequences shorter than 200 bp were excluded from the analysis. Sequences fulfilling these criteria were extracted and aligned against the Silva database using the same setting as described above. Only input sequences where at least 90% of alignments could be assigned to the genus *E. hallii* were considered positive hits.

### Metagenome Screening

The 152 stool metagenomic sequencing datasets created in the scope of the HMP were downloaded from ftp://public-ftp.hmpdacc.org/Illumina/stool/. The datasets contained 16.6 billion paired and unpaired Illumina sequenced reads. The smallest dataset contained 3 million sequences. The largest dataset contained 305 million sequences. On average datasets contained 109 million sequences.

Screening was performed using PduC, PduD, and PduE from the *E. hallii* DSM 3353 and *L. reuteri* JCM 1112 (PduCDE_DB). The RefSeq (Pruitt et al., 2007) protein (Version: August-10-2015) database served as additional database.

Data from the 152 datasets were aligned against the PduCDE_DB database using DIAMOND (Buchfink et al., 2015). Sequences that were successfully aligned in the previous step were extracted and realigned against the RefSeq database (Pruitt et al., 2007) using DIAMOND. The bit score of alignments of paired reads that mapped to the same reference sequence were boosted by 30%. Reads with the best scoring alignment to PduC, PduD or PduE of *E. hallii* or *L. reuteri* were included in analysis.

Ten datasets with in particular high (SRS017521, SRS011271, SRS018427, SRS050422, SRS015431) or low abundance (SRS048164, SRS013800, SRS019787, SRS016018, SRS016585) of *E. hallii* underwent a deeper metagenomic analysis. The full datasets were aligned against the RefSeq database using DIAMOND. Alignment scores were boosted if mate pairs aligned to the same reference. Results were then taxonomically classified using MEGAN’s lowest common ancestor algorithm in combination with the NCBI taxonomy (Huson et al., 2011). Functional classification was performed by extracting the best scoring alignment of each read and mapping it to the GI protein names.

## Results

### Growth, Substrate Utilization and Metabolite Formation of *E. hallii* in the Presence of Glucose, Lactate and Glycerol

We used two *E. hallii* isolates that are currently available from public culture collections which were both isolated from human stool samples: the type strain *E. hallii* DSM 3353 and the *E. hallii*-like isolate DSM 17630 (L2-7, Duncan et al., 2004). Both strains were grown in mYCFA medium containing various combinations of glucose, lactate, glycerol, and 1,2-PD (**Table 1**, **Supplementary Table S1**).

**FIGURE 2:**
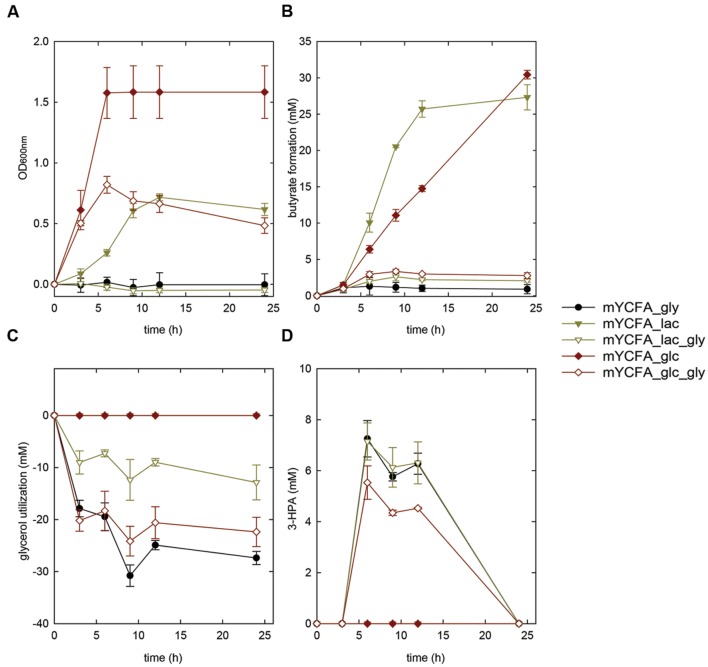
**Growth **(A)**, butyrate formation **(B)**, glycerol utilization **(C)**, and 3-HPA production **(D)** of *Eubacterium hallii* DSM 3353 in the presence of glycerol (mYCFA_gly), glucose (mYCFA_glc), glucose and glycerol (mYCFA_glc_gly), lactate (mYCFA_lac), and lactate and glycerol (mYCFA_lac_gly)**.

**FIGURE 3:**
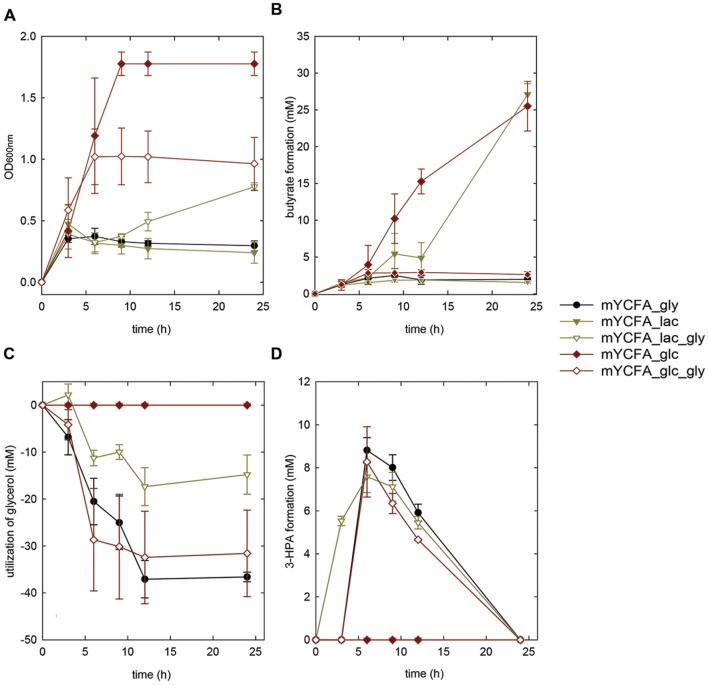
**Growth **(A)**, butyrate formation **(B)**, glycerol utilization **(C)**, and 3-HPA production **(D)** of *E. hallii* DSM 17630 in the presence of glycerol (mYCFA_gly), glucose (mYCFA_glc), glucose and glycerol (mYCFA_glc_gly), lactate (mYCFA_lac), and lactate and glycerol (mYCFA_lac_gly)**.

*Eubacterium hallii* reached highest OD (>1.5) when grown in the presence of glucose. *E. hallii* DSM 3353 and DSM 17630 used 32.8 ± 9 and 21.3 ± 5.1 mM of glucose supplied while producing equimolar amounts of butyrate (**Supplementary Table S2**, **Figures 2A,B and 3A,B**). When glycerol was added (mYCFA_glc_gly), growth of *E. hallii* was reduced and reached a maximum OD_600_ of about 0.7; little amounts of butyrate (<4 mM) were formed.

*Eubacterium hallii* DSM 3353 and DSM 17630 were also grown in the presence of lactate (mYCFA_lac), and of both lactate and glycerol (mYCFA_lac_gly). In mYCFA_lac, the lag phase was extended and the final OD_600_ of *E. hallii* was lower than in mYCFA_glc (**Figures 2A** and **3A**). The molar amount of butyrate produced was approximately half of the molar sum of lactate and acetate utilized (**Supplementary Table S3**). All lactate was utilized within 12 and 24 h of fermentation of *E. hallii* DSM 3353 and 17630, respectively, (**Supplementary Table S3**). The addition of glycerol to mYCFA containing lactate (mYCFA_lac_gly) also inhibited growth, lactate and acetate consumption (**Figures 2A** and **3A**, **Supplementary Table S3**).

When only glycerol was supplied instead of glucose (mYCFA_gly), *E. hallii* DSM 3353 did not grow while the OD_600_ of DSM 17630 increased by 0.35 units within 3 h of incubation (**Figures 2A and 3A**). The amount of butyrate produced was less than 3 mM for both strains and acetate was not utilized (**Figures 2B** and **3B**, **Supplementary Table S3**). Glycerol was converted to 3-HPA in all samples containing glycerol (**Figures 2C,D** and **3C,D**), irrespective of any other carbon sources present. Maximum 3-HPA concentrations were obtained after 6 h of incubation corresponding to 7 and 9 mM for *E. hallii* DSM 3353 and DSM 17630, respectively. Thereafter, the concentration of 3-HPA decreased steadily to undetectable levels. 1,3-PD was either not detected or present only at very low concentration of less than 1 mM throughout the incubations (data not shown). The reference compound for 3-hydroxypropionate, the alternative end product for glycerol metabolism (**Figure 1**; Gänzle, 2015) eluated as two peaks. As after growth of *E. hallii* in the presence of glycerol, no peaks at the rention times of the 3-hydroxypropionate standard appeared, it was assumed that 3-hydroxypropionate was not formed.

### Growth, Substrate Utilization and Metabolite Formation of *E. hallii* in the Presence of 1,2-PD

**FIGURE 4:**
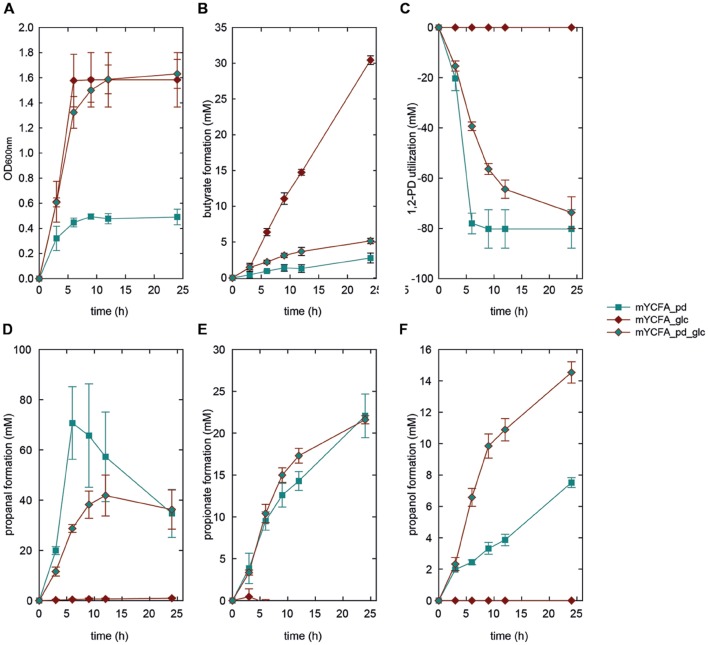
**Growth **(A)**, butyrate formation **(B)**, 1,2-PD utilization **(C)**, and propanal **(D)**, propionate **(E)**, and propanol **(F)** formation by *E. hallii* DSM 3353 in the presence of 1,2-PD (mYCFA_pd), glucose (mYCFA_glc), and glucose and 1,2-PD (mYCFA_pd_glc)**.

**FIGURE 5:**
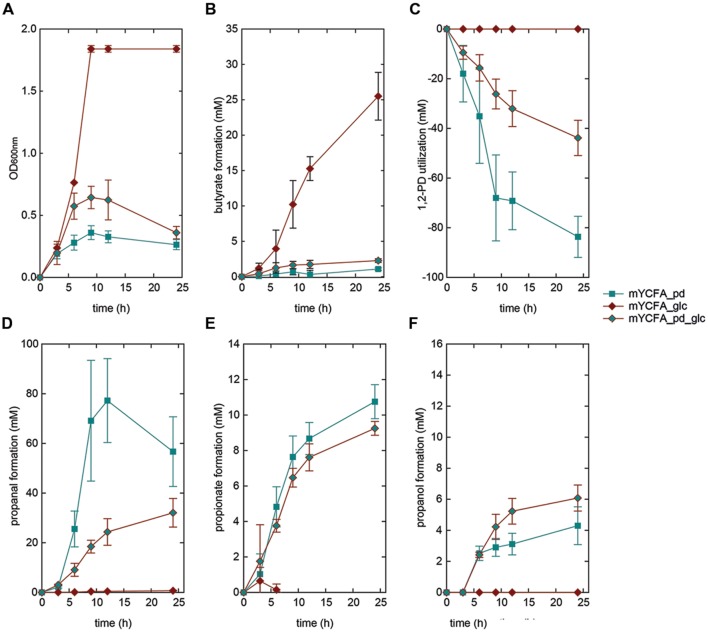
**Growth **(A)**, butyrate formation **(B)**, 1,2-PD utilization **(C)**, and propanal **(D)**, propionate **(E)**, and propanol **(F)** formation by *E. hallii* DSM 17630 in the presence of 1,2-PD (mYCFA_pd), glucose (mYCFA_glc), and glucose and 1,2-PD (mYCFA_pd_glc)**.

We also investigated the growth behavior of *E. hallii* in the sole presence of 1,2-PD (mYCFA_pd), and in combination with glucose (mYCFA_pd_glc). The presence of 1,2-PD impacted the growth of the two *E. hallii* strains differently: Growth of *E. hallii* DSM 3353 was similar in the presence and absence of 1,2-PD while the final optical density of *E. hallii* DSM 17630 was significantly reduced by the addition of 1,2-PD (**Figures 4A** and **5A**). In mYCFA_pd_glc neither strain used glucose, and butyrate was not produced (**Figures 4B** and **5B**, **Supplementary Table S2**).

**FIGURE 6:**
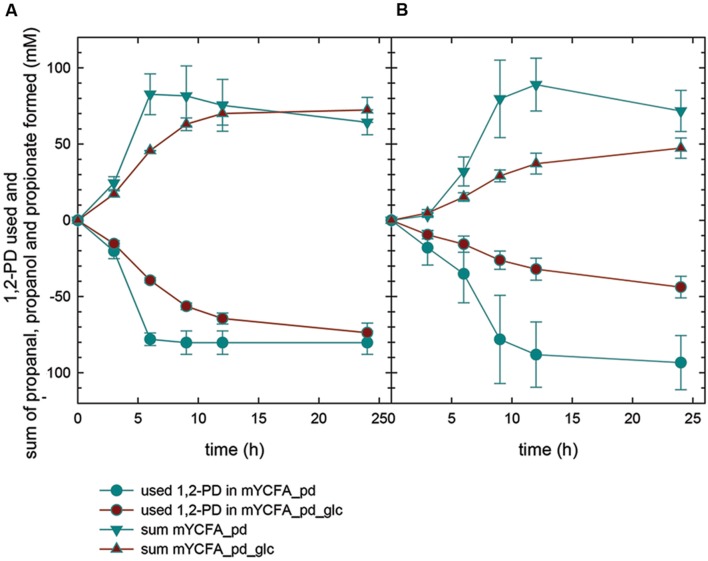
**1,2-PD utilization and sum of propanal, propionate and propanol formed by *E. hallii* DSM 3353 **(A)** and DSM 17630 **(B)****.

In mYCFA_pd, *E. hallii* DSM 3353 consumed all 1,2-PD within 9 h of incubation (**Figures 4C** and **5C**), resulting in the accumulation of propanal, propanol and propionate (**Figures 4** and **5D–F**). The molar yield of propanal, propanol and propionate added up to the molar amount of 1,2-PD used (**Figure 6**). Slower 1,2-PD utilization was detected when glucose was added (mYCFA_pd_glc) then without (mYCFA_pd). Hereby propanal did not accumulate and significantly (*p* < 0.05) more propanol was formed by *E. hallii* DSM 3353 (**Figure 4**). The utilization of 1,2-PD and corresponding metabolite formation was similar for *E. hallii* DSM 17630, however, the final amount of propanol produced was not significantly different in the absence or presence of glucose (**Figure 5**). *E. hallii* DSM 3353 and DSM 17630 produced about 20 and 10 mM of propionate, respectively, after 24 h of incubation, independently of the presence of glucose (**Figures 4E** and **5E**).

### Cobalamin Formation

The glycerol/diol dehydratase of *L. reuteri* is a cobalamin-dependent enzyme. *E. hallii* DSM 3353 possesses most of the genes necessary to form cobalamin (Engels et al., 2016; **Supplementary Figure S1**). To investigate whether *E. hallii* is able to synthesize cobalamin, a reporter strain assay employing cobalamin-auxotrophic *L. delbrueckii* subsp. *lactis* DSM 20355 as indicator strain was conducted. *L. delbrueckii* subsp. *lactis* grew after addition of cellular extracts of *L. reuteri* DSM 20016 and *L. rossiae* DSM 15814, which were used as positive controls. Addition of cell extracts of both *E. hallii* strains also resulted in growth of *L. delbrueckii* subsp. *lactis* (**Supplementary Figure S2**). Thus, we could confirm here that both strains of *E. hallii* are able to form cobalamin.

### MIC_50_ Values of Reuterin toward *E. hallii*

To determine the MIC_50_ values of reuterin toward *E. hallii*, both strains were grown in serial dilutions of reuterin in YCFA medium. OD_600_ values measured after 24 h of growth at 37°C were plotted against corresponding 3-HPA concentrations (**Supplementary Figures S3A,B**). The inflection points of the resulting curves were used to calculate MIC_50_ values. The MIC_50_ values of reuterin were determined to be 3.7 ± 0.2 mM (quantified as 3-HPA) for *E. hallii* DSM 3353 and also for *E. hallii* DSM 17630. In correspondence with these MIC_50_ values, visible growth of both strains was detectable in the presence of up to 7–8 mM 3-HPA (**Supplementary Figures S3A,B**).

### Abundance of *E. hallii* in 16S rRNA Gene Libraries and Metagenomes Derived from the HMP

We screened a total of 325 16S rRNA gene libraries obtained from the HMP for the occurrence and relative abundance of *E. hallii.* Sequences were aligned against the 16S rRNA gene of *E. hallii* strains DSM 17630 and DSM 3353. Successfully aligned sequences were then aligned against the Silva 16S rRNA database to remove false positives. *E. hallii* was detected in stool of 74% of the donors. Relative abundance ranged from 0 to 0.59% with a mean relative abundance of 0.044%. Most individuals harbored *E. hallii* with relative abundance of less than 0.1% (**Supplementary Figure S4**). We also used *pduCDE* as functional marker genes to screen 152 metagenomes derived from stool of adults in the scope of the HMP for the presence of *E. hallii* and *L. reuteri.* Sequences were aligned against a database that contained PduC, PduD, and PduE from *E. halli* and *L. reuteri* using DIAMOND. Successfully aligned sequences were then aligned against the RefSeq protein database in order to remove false positives. Depending on which Pdu subunit was investigated, occurrence ranged from 63 to 81% for *E. hallii* (**Table 2**). In comparison, *L. reuteri* was low abundant (0 to 2%) and PduC was only detected in 3 of 152 metagenomes.

**Table 2:** Occurrence and relative abundance of *Eubacterium hallii* and *Lactobacillus reuteri* PduCDE subunits in stool metagenomes obtained from HMP.

		Best hits	
	
*Pdu* subunit	PduE	PduD	PduC
***E. hallii***			
Occurrence	95/152 (63%)	103/152 (68%)	123/152 (81%)
Mean relative abundance	4 × 10^-6^	5 × 10^-6^	2 × 10^-5^
***L. reuteri***			
Occurrence	0	0	3/152 (2%)
Mean relative abundance	-	-	2 × 10^-8^


### Contribution of *E. hallii* to Glycerol/1,2-PD Metabolism in the Gut

**FIGURE 7:**
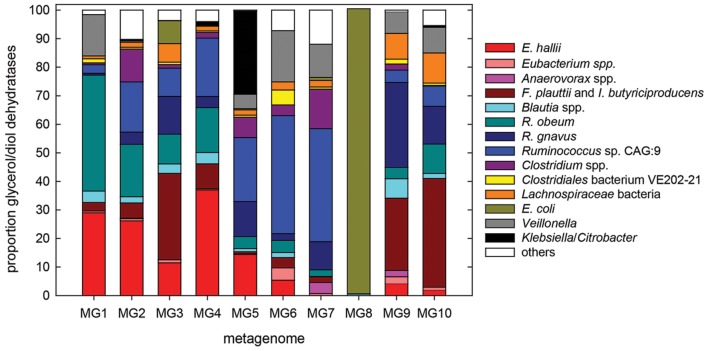
**Species contributing glycerol/diol dehydratases to the metagenomes of 10 individuals with high (MG1–MG5) and low (MG6–MG10) abundance of *E. hallii pduCDE;* others, groups all species with low hits**.

To further investigate to which extent *E. hallii* contributes to glycerol/1,2-PD metabolism, we determined species contributing glycerol/diol dehydratases using 10 metagenomes with high (MG1-5) or low (MG6-10) abundance of *E. hallii* PduCDE (**Figure 7**). In accordance to results obtained by screening for the presence of PduCDE, contribution of *E. hallii* to the entire glycerol/diol dehydratase pool was significantly higher (*p* < 0.05) in MG1-5 (23.6 ± 10.6%) than in MG6-10 (2.3 ± 2.4%). However, the relative abundance of all glycerol/diol dehydratases in metagenomes with high (MG1-5) or low (MG6-10) abundance of *E. hallii* was not significantly different (*p* > 0.05, log -3.2 ± 0.4 versus -4.7 ± 0.9 relative abundance). In 9 out of 10 metagenomes, *E. hallii*, *Ruminococcus obeum, R. gnavus*, *Ruminococcus* sp. CAG:9, *Flavonifractor prautii*, *Intestinimonas butyriciproducens*, and *Veillonella* spp. contributed more than 59% of all glycerol/diol dehydratases reads while in MG8, all glycerol/diol dehydratases reads were assigned to *Escherichia coli* (**Figure 7**). In general, a diverse pool of taxons potentially contributing to glycerol and 1,2-PD metabolism and thus propionate formation was identified.

### Distribution of Propionic Acid Formation Pathways

To investigate the contribution of the three major propionate formation pathways in the human gastrointestinal tract, we screened the previously mentioned 10 metagenomes for the relative abundance of genes encoding methylmalonyl-CoA decarboxylase Mmda, and lactoyl-CoA dehydratase Ldc, which are key enzymes in succinate and acrylate pathways (Reichardt et al., 2014), and compared to relative abundance of PduCDE of the propanediol pathway. There was no significant difference in relative abundance of key enzymes of the three propionate pathways between metagenomes with high or low abundance of *E. hallii.* Based on the relative abundance of the key proteins of all three pathways, the propanediol pathway contributed on average 31% to propionate formation, the succinate pathway 64%, and the acrylate pathway 5%.

## Discussion

*Eubacterium hallii* is a human gut microbe with a versatile utilization of carbon sources as it can grow and form butyrate using either glucose, or acetate and lactate as substrates.

Previous studies reported high abundance *E. hallii* in stool of 84 adults from Italy with 5.8, 4.8, and 3.2% in young adults, elderly and centenarians, respectively, determined using HitChip (Biagi et al., 2010). In contrast the stool of 9 out of 10 adults presumably from the UK contained between 0.03 and 2% (mean 0.34%) as determined using FISH (Hold et al., 2003). Relative abundance of *E. hallii* was 10–100 fold less in libraries derived from HMP. Despite this discrepancy, we observed high occurrence rates of *E. hallii.* Thus, *E. hallii* seems a regular constituent of an adult-like gut microbiota.

In contrast to *E. hallii, L. reuteri* was only rarely detected in adults. The ability to produce reuterin is conserved in strains of *L. reuteri* isolated from human feces (Frese et al., 2011). However, while being autochthonous in rodents and pigs forming biofilms at the non-glandular stratified squamous epithelium lining the upper gastrointestinal tract, *L. reuteri* occurs and persists only in the human gastrointestinal tract of some individuals (Walter, 2008), a finding that, we confirmed in this study.

When grown in the presence of glucose, both *E. hallii* strains produced equimolar amounts of butyrate. Molar amount of butyrate produced in mYCFA_lac was approximately half of the molar sum of lactate and acetate utilized; both findings are well in agreement with previous reports (Duncan et al., 2004). Glycerol is an abundant carbon source in the human intestine resulting from incomplete digestion and absorption of triglycerides, from luminal microbial fermentations, digestion of luminal fats, sloughed mucus and desquamated epithelial cells, as well as from intestinal clearing of endogenous plasma glycerol (Casas and Dobrogosz, 2000). According to Degnan et al. (2014a,b) an estimated 11% of gut microbes possess cobalamin-dependent glycerol/diol dehydratases and therefore have the genomic potential to synthesize reuterin from glycerol. The ecological role of reuterin formation remains to be elucidated. In growing cultures of *L. reuteri*, 3-HPA gets immediately reduced to 1,3-PD by a 1,3-PD dehydrogenase when glycerol and glucose are present to enable co-factor regeneration (Gänzle, 2015; **Figure 1**). Only during stationary phase in the absence of glucose, 3-HPA gets released from *L. reuteri* cells and accumulates in the medium (Vollenweider and Lacroix, 2004). For *E. hallii*, the formation of reuterin was observed whenever glycerol was present. The inconsistency of molar amounts of glycerol used and of 3-HPA detected during growth of *E. hallii* might be due to degradation of 3-HPA or its binding to proteinaceous compounds present in mYCFA and is well in agreement with previous observations (Luthi-Peng et al., 2002; Engels et al., 2016). Concentrations of about 7–8 mM (quantified as 3-HPA) completely inhibited visual growth and metabolism of the producer strain which is in agreement with growth inhibition observed in MIC_50_ assays. MICs of *E. hallii* were approximately 10-fold less than those observed for *L. reuteri* (30–50 mM) but were in the range of those reported for other *Eubacterium* spp. (Cleusix et al., 2007). In contrast to *L. reuteri*, metabolism of 3-HPA to 1,3-PD was not observed under the test conditions suggesting that *E. hallii* is not able to regenerate co-factors this way. Therefore glycerol metabolism and reuterin formation does not seem to enhance competitiveness of *E. hallii* in its ecological niche.

The other possible substrate of glycerol/diol dehydratases is 1,2-PD (**Figure 1**). 1,2-PD is derived from fermentation of fucose and rhamnose which are transformed by the activity of fucose/rhamnose isomerases, kinases and aldolases finally yielding L-lactaldehyde which can be further reduced to 1,2-PD by the activity of a propanediol oxidoreductase (Boronat and Aguilar, 1981). *E. hallii* does not harbor genes related to fucose and rhamnose utilization (genome ID: SAMN02415618). However, several gut microbes are capable of forming 1,2-PD (Saxena et al., 2010). Within the draft genome of *E. hallii*, we could identify homologs of *L. reuteri pduQ, pduP*, and *pduW* (**Supplementary Figure S1**) encoding enzymes which catalyze the transformation of propanal to propanol and propionate. There was, however, no homolog of *L. reuteri pduL* which presumably encodes the enzyme that transforms propionyl-CoA to propionyl phosphate. Nevertheless, as we observed the formation of propionate, it can be assumed that another enzyme is responsible for the phosphorylation. It can also be suggested that the metabolism of 1,2-PD to propionate increases the competitive advantage of *E. hallii* as this transformation generates one additional ATP (Gänzle, 2015).

*Eubacterium hallii* formed similar amounts of propionate in the presence or absence of glucose and did not utilize glucose if 1,2-PD was present. In contrast, butyrate formation from lactate only occurred when glucose was depleted (Duncan et al., 2004). *In vivo*, both pathways can be expected to happen as glucose is generally of low abundance in the colon. Due to its versatile metabolic activity, *E. hallii* seems to be able to impact the ratios of acetate and lactate and of butyrate and propionate. Both butyrate and propionate are important in gut microbiota/host homeostasis as they interact with the host epithelium and impact the immune system. Butyrate is a main energy source of colonocytes, it impacts cell proliferation and differentiation, and lowers the risk of colitis and colorectal cancer (Wong et al., 2006; Plöger et al., 2012). Propionate acts as a precursor for gluconeogenesis in the liver and also impacts cell differentiation with potential health promoting impact on intestinal inflammation, and cancer development (Reichardt et al., 2014).

In the intestine, acrylate, propanediol and succinate pathways lead to the production of propionate (Reichardt et al., 2014). The latter has been considered to contribute the most to propionate formation due to the widespread occurrence of the key gene Mmda encoding a methylmalonyl decarboxylase (Reichardt et al., 2014). Also here, the succinate pathway was identified as the most abundant propionate synthesizing pathway based on abundance of Mmda. However, the frequent detection of genes encoding glycerol/diol dehydratases suggests that 1,2-PD conversion contributes significantly to propionate formation, and that *E. hallii* is a relevant species in regard to intestinal propionate production. Among other species identified as glycerol and 1,2-PD utilizers by the activity of glycerol/diol dehydratases and therefore potential propionate producers were *R. obeum* and R. *gnavus, F. prautii* and *I. butyriciproducen*s, and *Veillonella* spp. *R. gnavus* is known to utilize mucins and degrades fucosylated glycans to propanol and propionate most likely via the formation of 1,2-PD (Crost et al., 2013). *F. prautii* and *I. butyriciproducens* of *Clostridium* cluster IV do not utilize fucose (Kläring et al., 2013). *Roseburia inulivorans* which utilizes fucose and degrades it to propionate and propanol (Scott et al., 2006), was not recovered in the screening conducted.

Glycerol/diol dehydratases are cobalamin dependent enzymes, and indeed, *E. hallii* produces cobalamin. Cobalamin formation is widespread among gut microbes and a variety of enzymes are cobalamin co-factor dependent (Degnan et al., 2014b). Nevertheless, the gut microbiota most likely does not supply cobalamin to the host in sufficient quantities as the main site of cobalamin formation is the colon whereas cobalamin specific receptors are located in the small intestine (Degnan et al., 2014b). Additionally, microbial competition for cobalamin is high, an estimated 80% of species requires cobalamin as co-factor, but only 25% can produce it (Degnan et al., 2014a). Considering this ecological context, *E. hallii* again gains competitive benefit within the gut microbiota as it is able to form cobalamin.

## Conclusion

We confirmed *E. hallii* as a common gut microbe with versatile substrate utilization spectrum. The ability to utilize glucose as well the glycan fermentation intermediates acetate, lactate and 1,2-PD to form butyrate or propionate points at *E. hallii* as a key species within the trophic interactions with the potential to highly impact the metabolic balance with final impact on gut microbiota/host homeostasis and host health.

## Author Contributions

Conceived and designed experiments: CE, H-JR, and CS. Performed experiments: CE, H-JR, and CS. Analyzed the data: CE, H-JR, and CS. Contributed reagents/materials/analysis tools: NB, CL. Wrote the paper: CE, H-JR, CS, and CL.

## Conflict of Interest Statement

The authors declare that the research was conducted in the absence of any commercial or financial relationships that could be construed as a potential conflict of interest.
