# Supplementary material for: The Common Gut Microbe Eubacterium hallii also Contributes to Intestinal Propionate Formation
Source: Front Microbiol. 2016 May 19;7:713. doi: 10.3389/fmicb.2016.00713 (PMC4871866; doi:10.3389/fmicb.2016.00713)
Supplement: Supplementary file 1 [file Data_Sheet_1.DOC]

Supplementary Data for

# The Common Gut Microbe *Eubacterium hallii* also Contributes to Intestinal Propionate Formation

Christina Engels^1#^, Hans-Joachim Ruscheweyh^2,3,4#^, Niko Beerenwinkel^2,4^, Christophe Lacroix^1^, Clarissa Schwab^1*^

^#^ Shared first authors

^1^ Laboratory of Food Biotechnology, Institute of Food, Nutrition and Health, Department of Health Sciences and Technology, ETH Zurich, Zurich, Switzerland

^2^ Department of Biosystems Science and Engineering, ETH Zurich, Basel, Switzerland

^3^ Research Informatics, Scientific IT Services, ETH Zurich, Basel, Switzerland

^4^ SIB Swiss Institute of Bioinformatics, Basel, Switzerland

^*^ *Corresponding author*: Clarissa Schwab

Content:

Supplementary Figures

Supplementary Tables

Supplementary References

**Figure S1.**


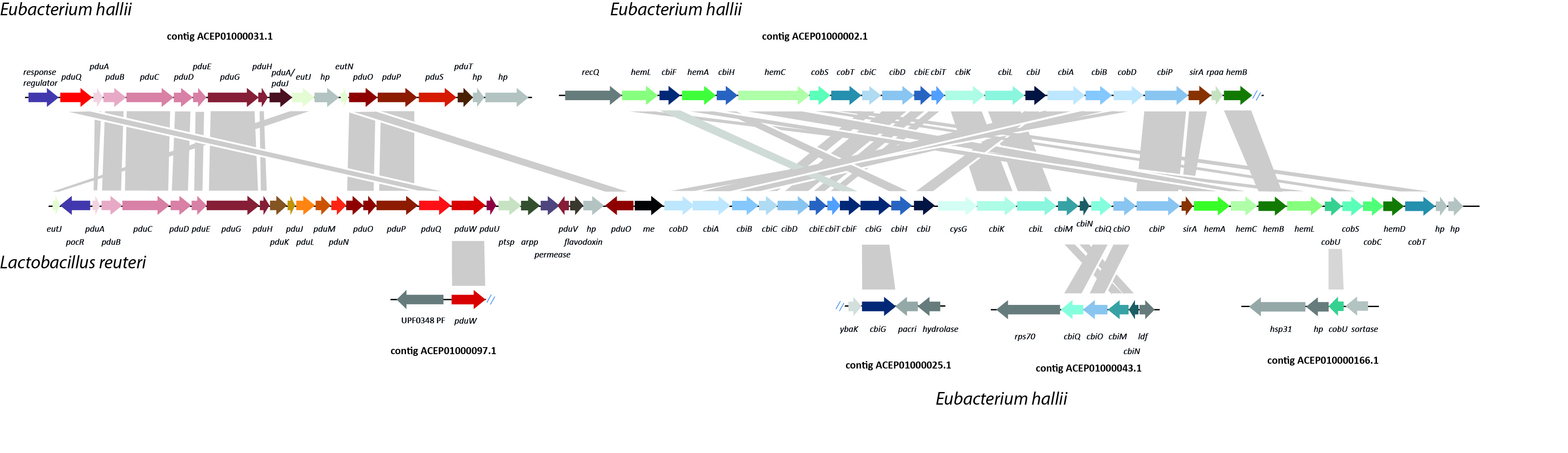


**Figure S1.** *Pdu-cob* locus of *E. hallii* DSM 3353 in comparison to *L. reuteri* (modified from Engels et al., 2016).

**Figure S2.**


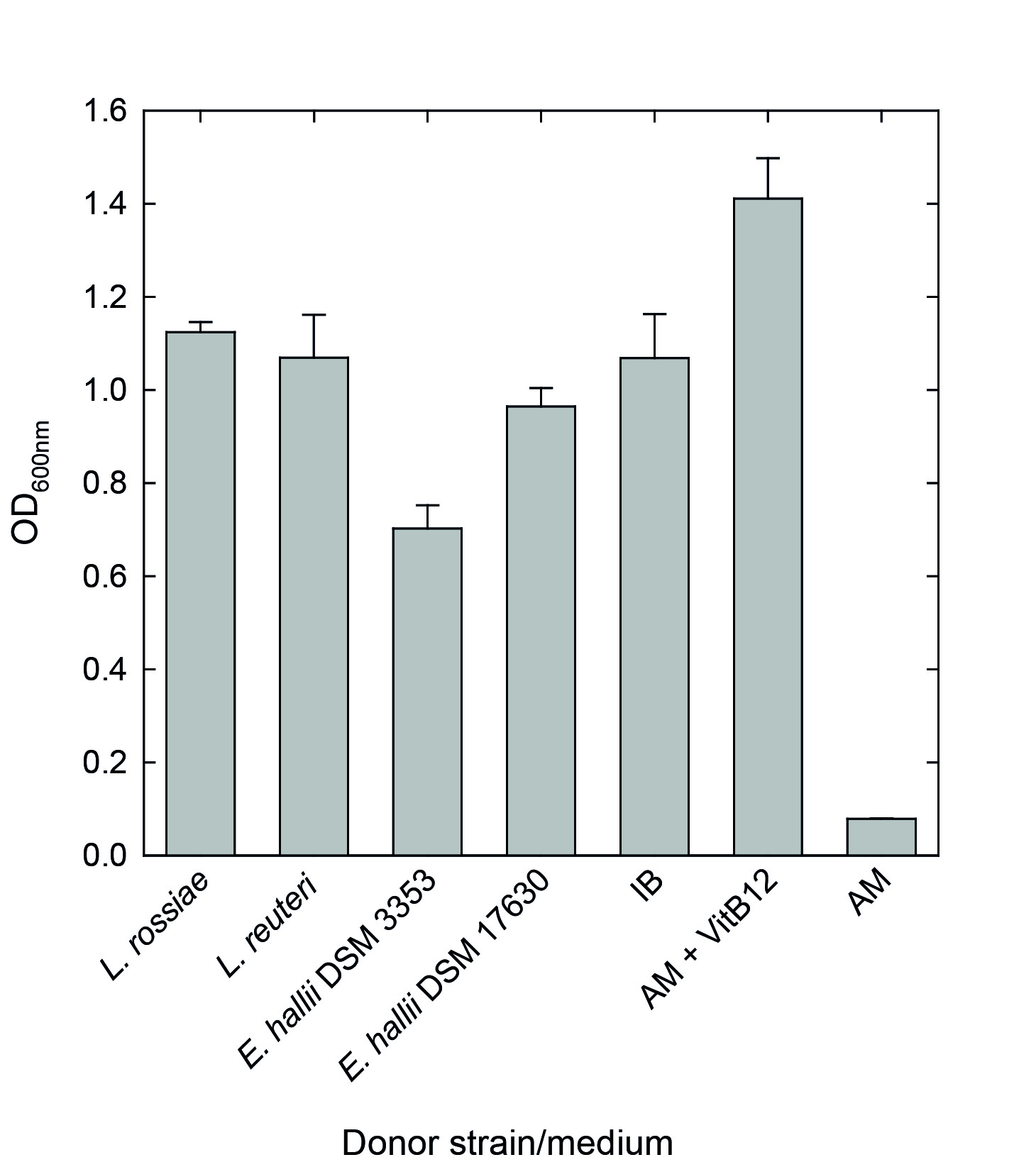


**Figure S2. Formation of cobalamin.** Shown are OD_600_ after 24 h of incubation of a vitamin B12-dependent indicator strain in the presence of *L. rossiae, L. reuteri, E. hallii* DSM 3353 and DSM 17630 crude cell extracts, in inoculation broth (IB), and in assay medium (AM) with or without additional vitamin B12 added.

**Figure S3.**

**
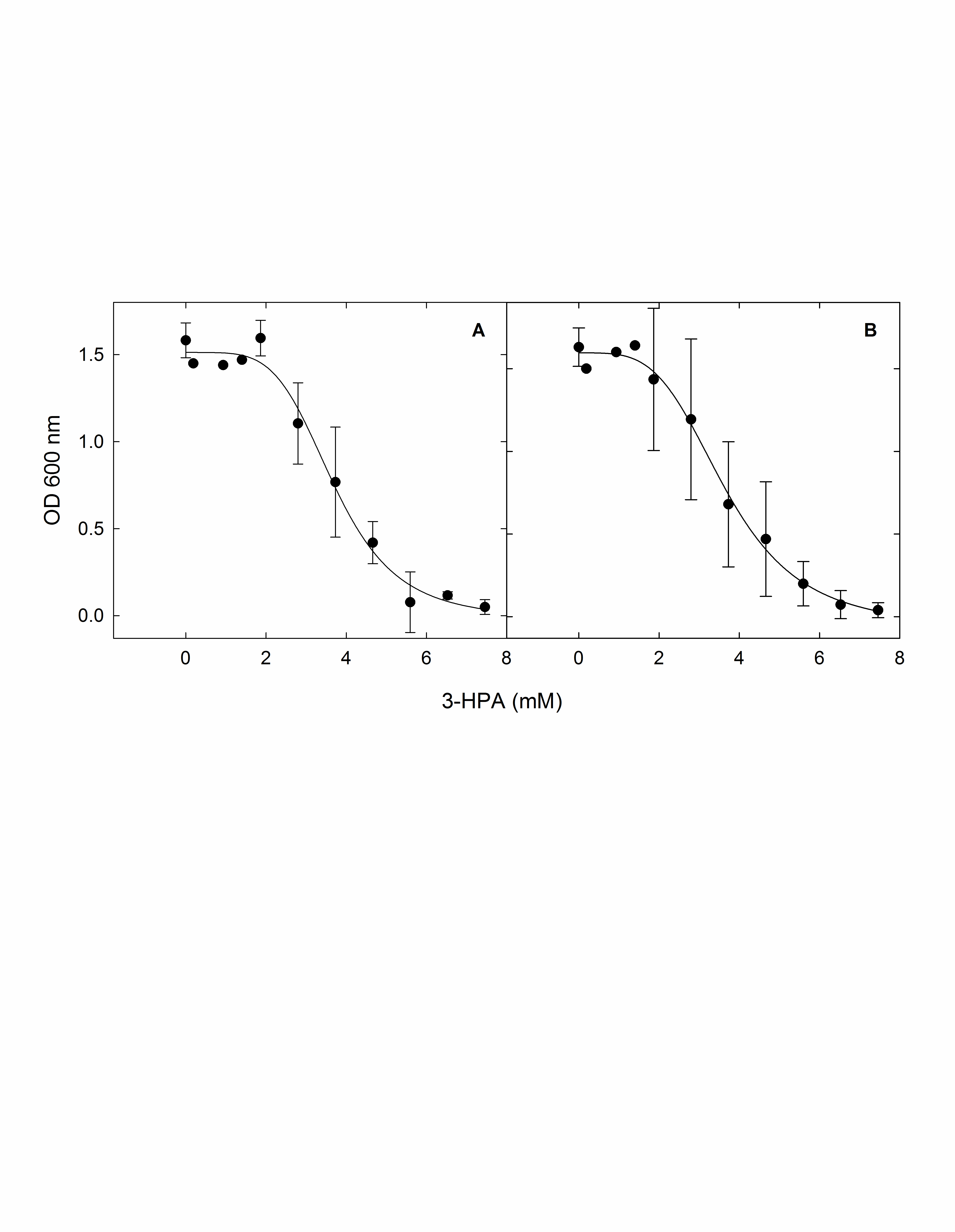
**

**Figure S3.** **Inhibition of *E. hallii* growth by reuterin.** Growth of *E. hallii* DSM 3353 (A) and DSM 17630 (B) in the presence of increasing concentrations of 3-HPA. Shown are mean values and standard deviations of four independent experiments.

**Figure S4.**

**Figure S4.** Number of *E. hallii* positive donors grouped in categories with mean relative abundance ranging from 0 to 0.6%. Samples were grouped stepwise dependent on relative abundance of *E. hallii* 16S rRNA genes in categories of 0.01% from 0 to 0.1%, and in categories of 0.1% from 0.1 to 0.6. Mean relative abundance of each group was calculated, and is shown here on the x-scale.

**Table S1.** Composition of mYCFA medium.

| **Component** | **Addition** |
| --- | --- |
| Amicase | 1% (w/v) |
| Yeast extract | 0.25% (w/v) |
| Sodium bicarbonate | 0.5% (w/v) |
| Glucose (replaced with various C-sources in modified YCFA, see Table 1) | 1% (w/v) |
| Mineral solution (3% (w/v) potassium dihydrogen phosphate, 6% (w/v) sodium chloride, 0.6% (w/v) magnesium sulfate, 0.6% calcium chloride (w/v)) | 15% (v/v) |
| Vitamin solution (0.01% (w/v) biotin, 0.01 (w/v) cobalamin, 0.03% p-aminobenzoic acid (w/v), 0.05% folic acid (w/v), 0.15% pyridoxamine (w/v). | 0.1% (v/v) |
| Volatile fatty acid mix (56.6% (v/v) acetic acid, 20% (v/v) butyric acid, 13.3% (v/v) propionic acid) | 0.31% (v/v) |
| Hemin (0.5 mg ml^-1^) | 0.02% |
| Resazurin (1 mg ml^-1^) | 0.1% |
| L-cysteine hydrochloride monohydrate | 0.1% |

**Table S2. Glucose balance. Glucose (mM) used during growth of *E. hallii* DSM 3353 and DSM 17630 in mYCFA containing glucose (mYCFA_glc), or propanediol and glucose (mYCFA_pd_glc).**

|  | **DSM 3353** | | | **DSM 17630** | | |
| --- | --- | --- | --- | --- | --- | --- |
| **Incubation (h)** | **mYCFA_glc** | **mYCFA_glc_gly** | **mYCFA_pd_glc** | **mYCFA_glc** | **mYCFA_glc_gly** | **mYCFA_pd_glc** |
| 0 | 0 | 0 | 0 | 0 | 0 | 0 |
| 3 | 0.6±4.4 | -3.2±0.9 | 0.5±0.9 | -5.3±5.0 | -1.1±1.0 | -1.4±1.4 |
| 6 | 2.8±6.4 | -3.7±3.3 | -0.2±1.5 | -3.6±6.2 | 0.8±1.6 | -0.5±1.4 |
| 9 | 0.3±7.6 | -3.5±3.9 | 0.1±1.5 | -4.1±5.0 | -1.4±2.8 | -0.5±0.6 |
| 12 | -8.5±6.8 | -1.0±0.8 | -0.2±1.6 | -9.7±5.1 | -4.2±5.2 | -0.8±1.6 |
| 24 | -32.8±8.8 | -6.7±3.9 | 0.6±0.7 | -21.3±5.1 | -2.0±8.6 | -1.7±0.3 |

**Table S3. Lactate and acetate balance of *E. hallii* DSM 3353 and DSM 17630 when grown in the presence of lactate.** Lactate and acetate used during growth of *E. hallii* DSM 3353 and DSM 17630 in mYCFA containing approximately 40 mM lactate (mYCFA_lac) or lactate and glycerol (mYCFA_lac_gly).

|  | **DSM 3353** |  |  |  | **DSM 17630** |  |  |  |
| --- | --- | --- | --- | --- | --- | --- | --- | --- |
| **Incubation (h)** | **mYCFA_lac** | | **mYCFA_lac_gly** | | **mYCFA_lac** | | **mYCFA_lac_gly** | |
| **Substrate** | **Lactate (mM)** | **Acetate (mM)** | **Lactate (mM)** | **Acetate (mM)** | **Lactate (mM)** | **Acetate (mM)** | **Lactate (mM)** | **Acetate (mM)** |
| 0 | 0 | 0 | 0 | 0 | 0 | 0 | 0 | 0 |
| 3 | -1.9±0.6 | -0.7±0.3 | 0.5±1.2 | 0.2±0.8 | 0.5±1.0 | 0.5±0.7 | 0.7±0.9 | 0±0.6 |
| 6 | -13.5±0.8 | -5.8±0.7 | -1.5±0.6 | -0.2±0.7 | -1.2±1.7 | -0.9±0.9 | 0.3±0.4 | -0.4±0.5 |
| 9 | -28.7±0.3 | -12.5±0.4 | 0.0±2.1 | 1.5±1.5 | -4.9±5.3 | -2.0±3.0 | 1.5±1.1 | 0.6±0.9 |
| 12 | -37.8±0.3 | -17.6±0.2 | -1.6±0.9 | 0.5±0.9 | -4.2±4.2 | -1.8±2.3 | 1.1±1.6 | 0.7±1.3 |
| 24 | -37.8±0.3 | -17.1±0.6 | -2.7±0.8 | -0.1±0.9 | -37.3±1.1 | -17.2±2.1 | -0.6±0.9 | -0.5±0.5 |

***Supplementary References***

Engels, C., Fekry, M.I., Zhang, J., Schwab, C., Lacroix, C., Sturla, S.J., and Chassard, C. (2016). The strict anaerobic gut microbe *Eubacterium hallii* transforms the dietary carcinogen amine 2-amino-1-methyl-6-phenylimidazo[4,5-b]pyridine (PhIP). *Environ. Microbiol. Rep.*Apr;8(2):201-9. doi: 10.1111/1758-2229.12369
